# Effectiveness of the head CT choice decision aid in parents of children with minor head trauma: study protocol for a multicenter randomized trial

**DOI:** 10.1186/1745-6215-15-253

**Published:** 2014-06-25

**Authors:** Erik P Hess, Kirk D Wyatt, Anupam B Kharbanda, Jeffrey P Louie, Peter S Dayan, Leah Tzimenatos, Sandra L Wootton-Gorges, James L Homme, Laurie Pencille RN, Annie LeBlanc, Jessica J Westphal, Kathy Shepel, Nilay D Shah, Megan Branda, Jeph Herrin, Victor M Montori, Nathan Kuppermann

**Affiliations:** 1 Knowledge and Evaluation Research Unit, Mayo Clinic, 200 First Street SW, 55905 Rochester, MN, USA; 2 Department of Emergency Medicine, Division of Emergency Medicine Research, Mayo Clinic, 200 First Street SW, 55905 Rochester, MN, USA; 3 Division of Health Care Policy and Research, Department of Health Sciences Research, Mayo Clinic, 200 First Street SW, 55905 Rochester, MN, USA; 4 Robert D and Patricia E Kern Center for the Science of Healthcare Delivery, Mayo Clinic, 200 First Street SW, 55902 Rochester, MN, USA; 5 Mayo Medical School, Mayo Clinic College of Medicine, 200 First Street SW, 55902 Rochester, MN, USA; 6 Children’s Hospitals and Clinics of Minnesota, 345 North Smith Avenue, 55102 St. Paul, MN, USA; 7 University of Minnesota Amplatz Children’s Hospital, 2450 Riverside Ave, 55455 Minneapolis, MN, USA; 8 Departments of Emergency Medicine and Pediatrics, Columbia University College of Physicians and Surgeons, 3959 Broadway CHN, 1-116 10032 New York, NY, USA; 9 Departments of Emergency Medicine and Pediatrics, University of California Davis School of Medicine, 2315 Stockton Boulevard, 95817 Davis, CA, USA; 10 Department of Radiology, University of California Davis School of Medicine, 4860 Y Street, Suite 3100, 95817 Davis, CA, USA; 11 Division of Pediatric Emergency Medicine, Departments of Emergency Medicine and Pediatrics, Mayo Clinic, 200 First Street SW, 55902 Rochester, MN, USA; 12 Parent Representative, Rochester, MN, USA; 13 Section of Creative Media, Mayo Clinic, 200 First Street SW, 55902 Rochester, MN, USA; 14 Department of Cardiology, Yale University School of Medicine, 330 Cedar St, Boardman 110, P.O. Box 208056, 06520-8056 New Haven, CT, USA; 15 Health Research and Educational Trust, 1 N Franklin St, 60606 Chicago, IL, USA; 16 Division of Endocrinology and Metabolism, Department of Internal Medicine, Mayo Clinic, 200 First Street SW, 55902 Rochester, MN, USA

## Abstract

**Background:**

Blunt head trauma is a common cause of death and disability in children worldwide. Cranial computed tomography (CT), the reference standard for the diagnosis of traumatic brain injury (TBI), exposes children to ionizing radiation which has been linked to the development of brain tumors, leukemia, and other cancers. We describe the methods used to develop and test the effectiveness of a decision aid to facilitate shared decision-making with parents regarding whether to obtain a head CT scan or to further observe their child at home.

**Methods/Design:**

This is a protocol for a multicenter clinician-level parallel randomized trial to compare an intervention group receiving a decision aid, ‘Head CT Choice’, to a control group receiving usual care. The trial will be conducted at five diverse emergency departments (EDs) in Minnesota and California. Clinicians will be randomized to decision aid or usual care. Parents visiting the ED with children who are less than 18-years-old, have experienced blunt head trauma within 24 hours, and have one or two risk factors for clinically-important TBI (ciTBI) from the Pediatric Emergency Care Applied Research Network head injury clinical prediction rules will be eligible for enrollment. We will measure the effect of Head CT Choice on: (1) parent knowledge regarding their child’s risk of ciTBI, the available diagnostic options, and the risks of radiation exposure associated with a cranial CT scan (primary outcome); (2) parent engagement in the decision-making process; (3) the degree of conflict parents experience related to feeling uninformed; (4) patient and clinician satisfaction with the decision made; (5) the rate of ciTBI at seven days; (6) the proportion of patients in whom a cranial CT scan is obtained; and (7) seven-day healthcare utilization. To capture these outcomes, we will administer parent and clinician surveys immediately after each clinical encounter, obtain video recordings of parent-clinician discussions, administer parent healthcare utilization diaries, analyze hospital billing records, review the electronic medical record, and conduct telephone follow-up.

**Discussion:**

This multicenter trial will robustly assess the effectiveness of a decision aid on patient-centered outcomes, safety, and healthcare utilization in parents of children with minor head trauma in five diverse EDs.

**Trial registration:**

ClinicalTrials.gov registration number: NCT02063087. Registration date February 13, 2014.

## Background

Blunt head trauma is a common cause of death and disability in children worldwide and accounts for approximately 52,000 deaths and 275,000 hospital admissions in the United States annually [1]. Each year more than 650,000 children visit emergency departments (EDs) in the United States with apparently minor blunt head trauma (Glasgow Coma Scale (GCS) scores of 14 to 15) [2].

A cranial computed tomography (CT) scan, the reference standard test for the emergent diagnosis of traumatic brain injury (TBI) in children with blunt head trauma, exposes children to ionizing radiation which has been linked to the development of brain tumors, leukemia, and other cancers [3,4]. Furthermore, although radiation exposure from a single head CT scan may not significantly increase an individual’s risk of cancer, patients often undergo multiple CT examinations over their lifetime and the cumulative radiation dose may exceed the 50 mSv [5] threshold that has been linked to the development of cancer among atomic bomb survivors [6].

One method to reduce CT use in patients with blunt head trauma is through evidence-based clinical prediction rules [7]. In 2009, Kuppermann *et al*. derived and validated two clinical prediction rules to accurately identify children at very low risk for clinically-important TBIs (ciTBIs) [2]. The rules were developed from a cohort of more than 42,000 children with minor blunt head trauma among 25 EDs in the United States. They have been externally validated [8] and are accurate for use in clinical practice [7]. Two rules were created, one for children younger than 2 years and a second for children between 2 and 18-years-old. The rules and study data allow for the classification of children into three risk groups: high risk, for whom a CT scan is recommended (risk of ciTBI for the younger than 2 and 2 to 18-years-old age groups is 4.4% and 4.3%, respectively); moderate risk, for whom observation or a CT scan are reasonable options depending on parental preference, clinician experience, and other factors (risk of ciTBI for the younger than 2 and 2 to 18-years-old age groups is 0.9% and 0.8%, respectively); and low risk, for whom a CT scan is typically not recommended (risk of ciTBI for the younger than 2 and 2 to 18-years-old age groups is <0.02% and <0.05%, respectively) [2]. Although the prediction rules recommend observation or a CT scan as reasonable options for children in the moderate risk group, the rules provide little guidance for clinicians regarding how to weigh each of these factors and engage parents in the decision-making process.

Shared decision-making (SDM) is a process of decision-making in medicine that has several key features: the involvement of the patient (or parent, in a child’s case) and the clinician, a sharing of information by both parties, and both parties taking steps to reach an agreement about which management option to implement [9]. While SDM may occur in a routine discussion during a clinical encounter, clinical decision-support tools may be necessary to effectively present comparative effectiveness research findings in a way that facilitates SDM. Decision aids provide the best scientific evidence available to support decision-making, prompt clarification of patient values and preferences, and have become increasingly used to provide the necessary decision support for SDM [10].

To facilitate SDM between clinicians and parents of children with minor head trauma at low-moderate risk for ciTBI, we sought to develop and test a decision aid, ‘Head CT Choice’. Specifically, we aimed to incorporate risk estimates for ciTBI from the Pediatric Emergency Care Applied Research Network (PECARN) prediction rules, along with the perspectives of parents and other stakeholders, in an iterative development process to produce a decision aid that is ready for testing. We hypothesized that use of the decision aid will significantly increase parents’ knowledge and satisfaction and safely tailor the rate of cranial CT scan and seven-day healthcare utilization to risk-informed parent preferences. In this manuscript, we describe the methods and protocol used to develop and refine the Head CT Choice decision aid and how we will test its effectiveness in a multicenter randomized trial.

## Methods/Design

### Study design

This practical [11] multicenter clinician-level parallel randomized trial compares an intervention group receiving a structured risk assessment and corresponding decision aid (Head CT Choice) to a control group receiving usual care. Institutional Review Board (IRB) approval has been obtained from the Mayo Clinic IRB (approval number: 13–004659), the University of Minnesota IRB (approval number: 1401 M46802), the Children’s Hospitals and Clinics of Minnesota IRB (approval number: 1312–117) and the University of California Davis IRB (approval number: 587396). The trial is registered at clinicaltrials.gov (registration number: NCT02063087).

### Study setting

The decision aid was developed and refined at the Mayo Clinic in Rochester, Minnesota (United States). The clinical trial will take place in five EDs in the United States: four in Minnesota and one in California: Mayo Clinic (an academic ED serving a largely rural population), University of Minnesota Amplatz Children’s Hospital (a university-based academic freestanding children’s hospital ED serving a largely urban population), Children’s Hospitals and Clinics of Minnesota Minneapolis (a private but academic freestanding children’s hospital ED serving a largely urban population), Children’s Hospitals and Clinics of Minnesota St. Paul (a private but academic freestanding children’s hospital ED serving a largely urban population), and University of California, Davis Medical Center (a university-based academic ED serving a largely urban population).

### Patient and stakeholder engagement in the trial

Parents and other key stakeholders were engaged early on in the design of this trial and will continue to be engaged throughout the entire investigation; they will comprise the Patient and Stakeholder Advisory Council (PSAC). The PSAC consists of an ED Patient Advisory Council (PAC) and a parent representative. The ED PAC consists of five individuals who have contributed the patient’s perspective to several practice and quality improvement initiatives at the Mayo Clinic in Rochester over the years - a Masters level ED quality specialist, three patients, and a nurse representative. The ED PAC reviewed and provided feedback on the study design and grant proposal and will continue to inform on the conduct of the trial, interpretation of the results, and dissemination of the study findings. The parent representative, who both provides her own perspective and serves as a liaison for the views of the ED PAC, has experience as a parent of a child with minor head trauma who was evaluated in the ED. The parent representative assisted in the development and iterative refinement of the Head CT Choice decision aid, provided feedback on the grant proposal submitted for funding, selected the primary outcome for the study, is an active member on the investigative steering committee, and participates in the study as a co-investigator.

### Eligibility criteria and participant identification

**Figure 1:**
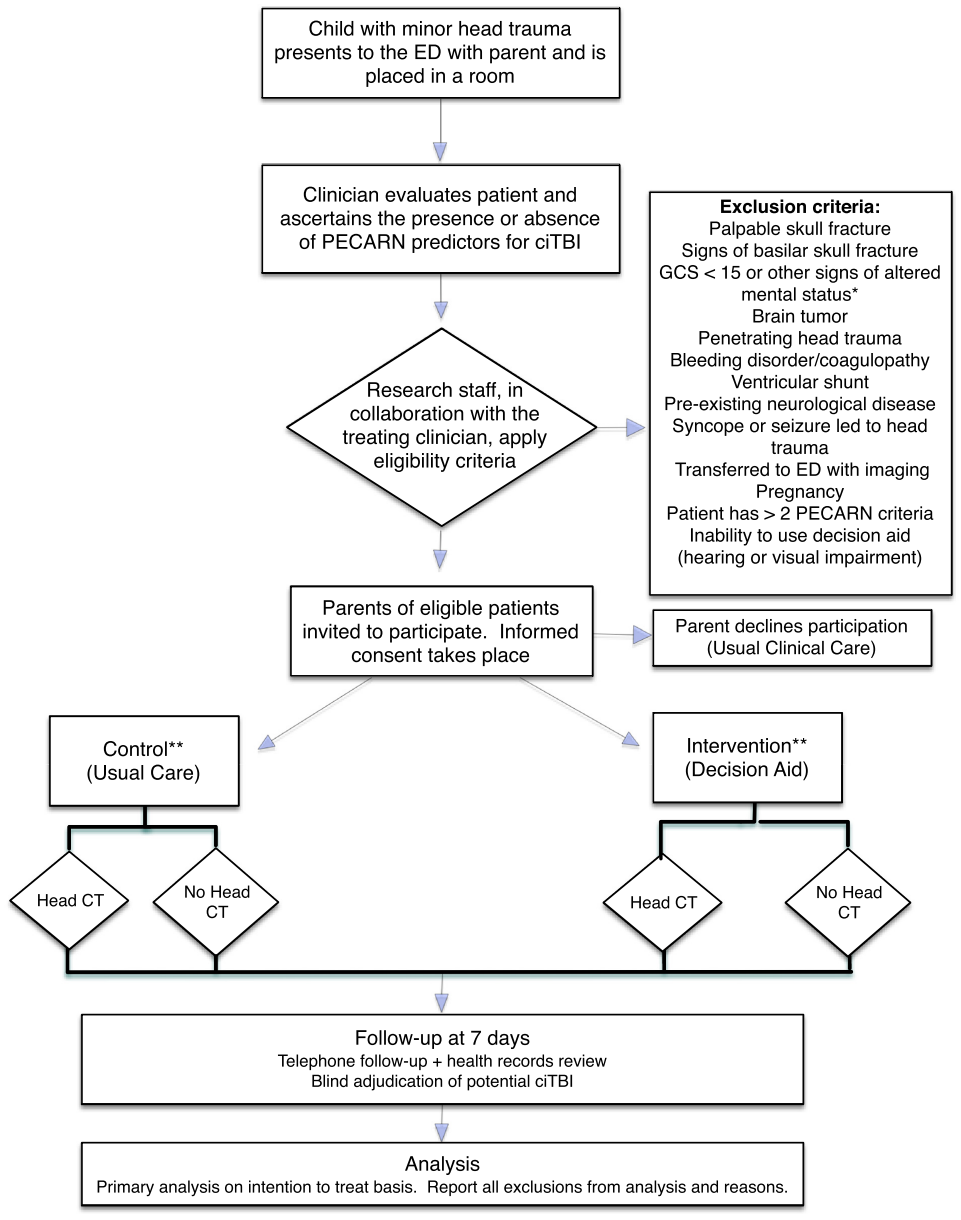
**Flow diagram showing patient identification and enrollment in the flow of patient care.** *Other signs of altered mental status include agitation, somnolence, repetitive questioning, or slow response to verbal communication. **Clinicians will be randomized to decision aid or usual care prior to patient enrollment. Parents will engage with their clinician according to the arm to which their clinician was randomized. ciTBI, clinically-important traumatic brain injury; CT, computed tomography; ED, emergency department; GCS, Glasgow Coma Scale; PECARN, Pediatric Emergency Care Applied Research Network.

Eligible participants will be parents seeking care for a child or adolescent who is less than 18-years-old, has experienced blunt head trauma (above the eyebrows and not isolated to face or eyes) within 24 hours, and has at least one of the PECARN risk factors for ciTBI (except for the two higher risk variables of altered mental status or signs of skull fracture) [2]. Eligible clinicians will be attending physicians, fellows, and midlevel providers caring for children with minor head trauma. Children with signs of skull fracture, GCS < 15 or other signs of altered mental status, brain tumor, penetrating head trauma, bleeding disorder or coagulopathy, ventricular shunt, pre-existing neurological disease, syncope or seizure that preceded the head trauma, transferred to the ED with imaging already obtained, known pregnancy, or > 2 PECARN risk factors will be excluded. Figure 1 displays the process of patient identification and enrollment that will be used in the trial.

### Randomization

Given the risk of contamination when randomizing at the patient level, we will randomize at the clinician level. Randomizing at the clinician level will obviate the risk of contamination associated with randomizing at the patient level. To decrease the risk of contamination by the sharing of information regarding the intervention between clinicians, we will emphasize to participating clinicians the importance of not sharing information about the intervention with their colleagues during the enrollment period. In addition, study coordinators will monitor for contamination at the time of patient enrollment and through review of the video recordings of clinician-patient discussions. Clinicians will be dynamically randomized and stratified [12] by site and by whether their primary clinical training is in a pediatric specialty (pediatrics or pediatric emergency medicine) or another specialty (such as general emergency medicine, family practice, or internal medicine). Informed consent to participate in the study and have their clinical encounters audio and video recorded will be obtained from clinicians at each site prior to enrollment. After clinician consent is obtained, a statistician will centrally randomize the clinician and communicate the study arm assignment to the site principle investigator and study coordinator. This centralized process will effectively conceal allocation prior to randomization.

### Participant identification and recruitment

Study coordinators will identify potentially eligible parent/patient dyads based on a chief complaint related to head trauma recorded at the time of ED registration and communication with treating clinicians in real-time. Senior resident physicians who often first evaluate the patient in the settings in which the trial will be conducted will be instructed to obtain the PECARN risk factors when conducting the initial history and physical examination and to not discuss the diagnostic options with the parent (Figure 1). Once these data have been obtained, the clinical predictors will be communicated to the study coordinator to determine if the patient has at least one of the (non-high-risk) PECARN predictors and meets all other eligibility criteria. The study coordinator will record these data on the eligibility assessment case report form (Additional file 1). Once patient eligibility is confirmed, study coordinators will obtain written informed consent from the parent(s) and assent from the patient to participate in the study and video record the discussion with their clinician.

### Intervention arm

#### ***Development and refinement of the decision aid***

A multidisciplinary research team including clinicians, health service researchers, a graphic designer, a radiation physicist, and patient representatives collaboratively developed the initial Head CT Choice decision aid. Using methods we have employed to develop other decision aids [13-17], we designed and refined Head CT Choice through an iterative process that involved design of an initial prototype, refinement based on the feedback received, use of the prototype in simulated parent/patient dyads in the ED setting, and use in ED clinical encounters in the flow of patient care [18].

In the original PECARN minor blunt head trauma study, only a summary estimate of the risk of ciTBI in the moderate risk group (0.9% for younger than two years and 0.8% for two years and older) was reported in the published manuscript [2]. To maximize the granularity of risk estimates provided to parents, we obtained the PECARN public use database [19] and calculated the precise risk of ciTBI based on the presence of any (non-high-risk) isolated PECARN risk factor (such as vomiting) or the combination of up to two simultaneous risk factors (such as vomiting and loss of consciousness). For patients whose physicians are randomized to the intervention arm, the clinician will be shown the specific risk estimate for their patient, including the exact proportion and 95% CI. In the case of a patient with two risk factors, the clinician will be shown the risk estimate, exact proportion, and 95% CI for each isolated risk factor and for the combination of the two risk factors. Patients with more than two PECARN risk factors (in addition to those with either of the high risk factors) will be excluded from enrollment due to the very few numbers and the higher risk for these patients. In the trial, coordinators will select the decision aid that corresponds to the risk for ciTBI of that isolated risk factor or, in the case of two risk factors, the highest risk estimate displayed.

In the process of decision aid development and refinement the following themes consistently emerged: (1) clarifying the differences between concussion and TBI, given recent social media attention on concussion; (2) communication of individual patient risk using natural frequencies, a consistent denominator, a prose description of the natural frequencies, and pictographs; (3) limiting the amount of information to that which is relevant to the decision at hand and best facilitates a conversation between the parent and clinician without undue focus on the decision aid itself; and (4) clearly outlining the choice of a head CT scan during the ED visit, home observation if no CT scan is obtained, or having the clinician make the decision on the parent’s behalf.

The Head CT Choice decision aid (Additional file 2) is a three-page paper tool that takes into account each of these key themes in numerically ordered sections to facilitate dialogue between the clinician and the parent. The decision aid first graphically highlights key differences between concussions and other types of significant brain injuries and communicates the child’s risk for ciTBI. The second page clearly outlines the decision that is to be made (head CT scan in the ED versus home observation) and educates the parent on the concerning signs and symptoms of TBI and the indications for return visit to the ED should they opt for home observation without a CT scan. Next, a table compares the key advantages and disadvantages of each management option and invites the parent to circle the issues that are most important to them. Finally, the decision aid outlines the three management options: head CT scan in the ED versus further observation at home with no CT scan, or having the ED clinician decide on the parent’s behalf. It also includes check boxes to document the decision along with a reminder that they will have the opportunity to revisit the decision with their clinician while in the ED. The third and final page includes a space to provide a contact phone number should the parent need to contact a healthcare provider after leaving the ED.

#### ***Training***

Participating clinicians at each study site were invited to attend a one-hour grand rounds lecture on the use of the decision aid, which included a demonstration of the tool. Clinicians randomized to the intervention arm will also be provided with brief video clips demonstrating use of the decision aid, which site principal investigators will review with the participating clinicians and will also be available for review at their convenience. Study staff will also be available at the point-of-care to provide brief, individualized re-training to clinicians on an as-needed basis. They will also offer reminders of how to use the decision aid when deviations in the quality of delivery are observed in real-time or reviewed on the video recordings of the clinical encounter.

#### ***Usual care***

For parent/patient dyads cared for by clinicians randomized to the usual care arm, the clinician will discuss the management options with the parent in that clinician’s usual fashion. The clinician will not be given access to the decision aid. The patient’s risk will be calculated for study purposes, however, the patient and clinician will be blinded to that patient’s precise risk of ciTBI beyond that which is available in the peer-reviewed literature [2]. As in the intervention arm, the clinician-patient management discussion will be video and audio recorded to assess the degree to which the clinician engages the parent in the decision-making process and to monitor for contamination in the control arm.

#### ***Parent and patient characteristics***

We will assess parent’s preferred degree of involvement in medical decision-making, literacy, and numeracy (Additional file 3). Parent’s preferred degree of involvement in decisions about medical treatment will be assessed using the control preferences scale [20], a validated scale that contains five pictorial representations accompanied by a statement that depicts a continuum of styles ranging from the patient making the decision independently, to the patient and clinician making a shared decision, to the clinician making the decision independently. Patients will be categorized with regards to health literacy in the following fashion: their literacy will be considered to be inadequate if they respond to the specific questions as either ‘not at all’ or ‘somewhat’ for confidence in completing medical forms. Otherwise, they will be considered to have adequate health literacy. Parent subjective numeracy will be assessed immediately before use of the decision aid using the validated Subjective Numeracy Scale [21,22]. The scale is an eight-item questionnaire that assesses parents’ comfort in working with numbers on a six-point Likert scale. Parent demographics, including the highest level of education and household income, will also be obtained by self-report at the time of enrollment.

Detailed patient characteristics will also be collected at the time of enrollment, including age, sex, race, ethnicity, GCS score and the presence or absence of each of the PECARN predictors at the time of enrollment. Additionally, we will collect data on the course of the patient’s symptoms in the ED (whether headache, vomiting, or mental status changes developed or improved), whether there were any concomitant substantial non-head injuries diagnosed, how long the patient was observed in the ED, whether a head CT scan or other imaging was obtained, any findings on the head CT scan, ED length of stay, and the patient’s type of departure from the ED (home or hospital admission).

#### ***Clinician characteristics***

A study coordinator will identify the primary clinician caring for the patient at the time of enrollment and collect data regarding whether they are a faculty physician, fellow, or advanced practice provider (nurse practitioner or physician assistant). The faculty physicians’ and fellows’ specialty training will also be collected (pediatrics, pediatric emergency medicine, general emergency medicine, family medicine, or internal medicine).

#### ***Outcome measures***

The outcomes were selected based on input from key stakeholders, including parent representatives, practicing clinicians, researchers (including shared decision-making experts), and health policy decision makers. In discussions with patient representatives it became clear that knowledge of the risk of ciTBI and the advantages and disadvantages of a head CT scan versus home observation were of greatest importance and so parent knowledge was selected as the primary outcome. All other outcomes will be secondary outcomes.

### Parent outcomes

#### ***Parent knowledge***

We will assess parent knowledge by means of a post-visit survey administered immediately after the clinical encounter (Additional file 4). Eleven questions will assess parents’ knowledge regarding concussion, their child’s individual risk of ciTBI, the available diagnostic options, the risks related to radiation exposure associated with a head CT scan, the potential for a CT scan to identify incidental abnormalities that may require further investigation, and reasons to return to the ED for re-evaluation should their child’s symptoms or signs worsen after ED discharge. We will measure the percent of knowledge questions answered correctly and determine the mean difference between knowledge scores for each study arm.

#### ***Parent satisfaction***

We will measure parental satisfaction in the way information was shared during the encounter by asking five questions using a seven-point Likert scale (Additional file 4). For the analysis, we will classify satisfaction into satisfied/very satisfied versus other responses.

#### ***Parent engagement in the decision-making process***

We will measure the degree to which clinicians engage parents in the decision-making process using the validated Observing Patient Involvement in Decision-Making (OPTION) scale [23,24]. The OPTION scale is an instrument in which an independent observer views a video recording of the clinician-patient interaction and measures the extent to which a clinician exhibits twelve behaviors on a four-point scale. OPTION scores are transposed to a 0 to 100 scale for interpretation and analysis, where higher scores indicate greater clinician involvement of the patient. A proportion of encounters will be calibrated in duplicate to ensure the reproducibility of measurements.

#### ***Decisional conflict***

We will measure the degree of conflict parents experience using the validated decisional conflict scale (DCS) (Additional file 4) [25,26]. The 16 items of the DCS are scored on a 0 to 4 scale; the items are summed, divided by 16, and then multiplied by 25. The scale is from 0 to 100, where higher scores are reflective of parental uncertainty about the choice.

#### ***Trust in the physician***

We will measure parents’ trust in their clinician using the validated Trust in Physician Scale (Additional file 4) [27,28]. There are 11 items with a scale of 1 to 5; the items are summed, divided by 11, and then multiplied by 100. The scale ranges from 0 to 100, where higher values are reflective of higher levels of trust in their physician.

### Patient outcomes

#### ***Safety***

We will assess patient safety by comparing the rate of ciTBI in each study arm. We will define ciTBI as we did in a prior study [2]: death from TBI, intubation for more than 24 hours for TBI, a neurosurgical procedure, or hospital admission of two or more nights for the head injury in association with TBI on a CT scan. Site investigators, blinded to ED data, will verify outcomes by health record review. CT scans will be obtained at the ED clinicians’ discretion and interpreted by site faculty radiologists. For outcome classification, a study pediatric radiologist unaware of the arm to which the patient was randomized will make definitive interpretations of inconclusive CT scans. Study coordinators will contact parents of children with minor head trauma by telephone starting at seven days after the index ED visit to ensure no outcomes are missed. Data from two cohorts (with a combined total of more than 59,000 children with minor head trauma [2,29]) indicate that no cases of clinical deterioration due to delayed intracranial hemorrhage occurred after 72 hours from the initial trauma, so we are confident that the seven-day follow-up interval will capture all potential adverse events.

#### ***Proportion of patients who undergo cranial CT scans***

The decision to obtain a CT scan or not is the most immediate clinical decision related to use of the decision aid. The study coordinator enrolling the patient will ascertain this in real-time and confirm the accuracy of the data by health record review.

#### ***Healthcare utilization***

We will assess healthcare utilization for the seven days following the ED visit. Healthcare utilization will include measures such as hospitalization, re-hospitalization, primary and specialty healthcare visits, and diagnostics including CT scan and magnetic resonance imaging (MRI) use. These measures will be obtained by health record review, review of itemized hospital charges on the Universal Billing form-92 and Universal Billing form-04 forms (summary billing statements), and parental report at the time of the seven-day follow-up telephone call. To assist parents in collecting utilization data, we will provide a form (Additional file 5) to document this information at the time of discharge from the ED or the hospital. This will allow for a more standardized collection of these data elements if parents receive follow-up care at a location other than the primary institution.

### Clinician outcomes

#### ***Satisfaction***

Clinician satisfaction will be assessed immediately after the ED visit via a questionnaire regarding the helpfulness of the decision aid and the clinician’s satisfaction with the way information was shared on a seven-point Likert scale (Additional file 6). For the analysis, we will classify satisfaction into satisfied/very satisfied versus other responses.

#### ***Preference***

We will assess clinicians’ preferred decision-making style immediately after the visit using the validated control preference scale (Additional file 6) [20]. Frequencies and counts of each pictorial selected by clinicians will be reported along with correlation to the patients’ selected pictorials from the control preference scale.

### Statistical considerations

#### Sample size

Accounting for a lost to follow-up rate as high as 5% (based on a 2% rate we observed in our previous ED SDM trial) [14], we anticipate enrolling and obtaining outcomes on approximately 950 patients over the study period with a 1:1 ratio between the intervention and control arms. Rather than assume that patient outcomes would be independent of the clinician treating them, we assumed an intra-clinician correlation of *P* = 0.05 to adjust for the clustering of patients when estimating statistical power [30]. Under this assumption, the anticipated number of patients will provide the degree of power to detect differences in each of the patient and stakeholder-important outcomes as specified in Table 1, using two-tailed tests and α = 0.05.

**Table 1:** Sample size calculations

**Outcome (n = 950)**	Usual care*	Decision aid*	**Difference**	**Power**
**Parent knowledge**	44%	60%	16%	>99%
**Parent engagement in the decision-making process**	7.0 (5.5)	27.0 (8.2)	20.0	>99%
**Decisional conflict†**	36 (19)	21 (21)	15	>99%
**Trust in the physician**	79 (20.0)	84 (20.0)	4.1	86%
**Parent satisfaction with the decision made (% agree or strongly agree they are satisfied)**	70.0 (26)	80 (26.0)	10.0	>99%
**Safety (clinically-important traumatic brain injury)‡**	0.9%	0.9%	0%	82.5%
**Proportion of children who undergo head CT§**	49%	34%	15%	95%
**Healthcare utilization**	8.3 (0.8)	6.8 (0.7)	1.5	>99%

*Estimates were determined from our completed randomized trial for adult patients presenting to the emergency department with chest pain and are presented as mean (standard deviation) or percentage.

†Lower decision conflict scores indicate less conflict experienced by patients related to feeling uninformed.

‡ Non-inferiority, one-sided test with alpha = 0.05 with a maximum difference of 2%. Baseline rates determined from the PECARN prediction rule study.

§ Obtained from the rate of CT scan in the moderate risk group of patients in the PECARN prediction rule study.

CT, computed tomography.

#### ***Analysis plan***

We will conduct the study according to the intention-to-treat principle, including all parent/patient dyads in the arm to which they were randomized, regardless of whether they received the intervention assigned. We will adhere to the CONSORT guidelines [31] to transparently report study results and ensure that sufficient information is included to allow for assessment of the study’s internal and external validity.

We will compare outcomes between study arms using t-tests for continuous outcomes and *χ*^2^ tests for dichotomous outcomes, adjusted for clustering by clinician and stratified by study site [30]. If there are differences in baseline characteristics between the two study groups, these will be accounted for using hierarchical generalized logistic or linear regression models that include an indicator for study arm [32].

For the assessment of healthcare utilization, we will compare utilization descriptively and using multivariable models, using two-part or one-part generalized linear regression models. We will use the Park test to assess the appropriate distribution, and use a two-part model for outcomes where more than 10% of the subjects have zero outcome measures. We will also analyze the results using a subset of patients who primarily receive all of their healthcare at the participating centers. We expect this will provide a sensitivity analysis of the overall trial results based on self-reporting.

We will perform descriptive analyses to describe any potential heterogeneity of treatment effect (HTE) and facilitate synthesis of subgroup results in future meta-analyses. We will conduct descriptive HTE analyses by age, gender, parent race/ethnicity, and highest attained parental education level. The outcomes assessed with HTE analyses will be the same as those assessed in the trial. We will also conduct interaction testing to determine the interaction between the decision aid and each pre-specified patient characteristic.

#### ***Missing data***

We will make every effort to minimize the occurrence of missing data. Items self-reported by parents at the seven-day follow-up telephone call will attempt to be verified (or ascertained, if missing) by medical record and healthcare billing review. A study analyst will conduct frequency reports every two weeks to identify and obtain missing data. Patients with missing outcome data will not be included in the assessment of that outcome. Rates of missing data will be reported as well as known reasons for missing data. For data used to adjust study comparisons, we will use multiple imputation to account for any data that are missing at random.

#### ***Safety and monitoring***

An independent data and safety monitoring board (DSMB) will monitor safety, scientific, and ethical aspects of the study. The DSMB has already met (prior to beginning enrollment) and will meet every six months throughout the duration of the study. Though every effort will be made to minimize post-randomization exclusions, patients will be excluded if they are found to meet exclusion criteria that were not recognized at the time of enrollment or they choose to withdraw [33]. Any potential ciTBIs following discharge from the ED or hospital will be reported to the principal investigator and the DSMB. The authority to stop the study will be retained by the DSMB and must be based on a consensus. The study will not be monitored for early termination due to benefit or futility.

## Discussion

We have described the methods employed to develop and test the first decision aid for minor blunt head trauma in children; Head CT Choice. With the decision aid developed, we will conduct a multicenter clinician-level parallel randomized trial to measure the effectiveness of the decision aid on patient-centered outcome measures, safety, and healthcare utilization in parents of children with minor head trauma. In the context of prior and ongoing work on the development and implementation of the PECARN clinical prediction rules, the current study informs and expands on the incorporation of parental preferences, clinician experience, and other factors that influence the decision of whether to obtain a head CT scan in children at moderate risk for ciTBI. We anticipate that the development and testing of a decision aid for use in this moderate risk group will equip clinicians with the tools needed to educate, empower, and engage parents in the decision-making process. Use of this aid will also facilitate risk communication, thus strengthening the doctor-patient relationship in the context of an emergency care interaction limited by time, the lack of continuity of care, and the need for clinicians to provide care for multiple patients simultaneously.

Given that we are randomizing at the clinician level, there is some risk of contamination if clinicians randomized to usual care get access to the decision aid and gain experience with using it in clinical encounters with parents of children with minor head trauma. We will take several steps to minimize the risk of contamination. First, we will emphasize to clinician participants the importance of not sharing the decision aid with their colleagues. We will also have study coordinators monitor for this in the process of conducting the trial. Second, using the PECARN public access dataset, we have generated a calculator that provides individual risk estimates for each of the isolated PECARN risk factors and for any two risk factors combined, in addition to the exact proportions and 95% CI’s. We have verified the accuracy of these risk estimates with the principal investigator of the PECARN head trauma study (NK), and these estimates are not accessible to clinicians at the point-of-care. Third, only the site coordinators will have access to copies of the decision aid and clinicians will not be provided a copy of the decision aid in the usual care arm. Fourth, site coordinators will be carefully trained to confirm the accuracy of each of the PECARN variables and to obtain sign-off from the treating clinician, but to keep the clinician blinded to the risk estimate for ciTBI unless that clinician is randomized to the decision aid. Fifth, we will make every effort to obtain video and audio data of the discussion between the clinician and the patient regarding whether to obtain a head CT scan during the ED visit or to further observe their child at home in both the intervention and control arms, and we will monitor for contamination by review of these data. Finally, if contamination does occur, we anticipate this will bias the effect estimates in each arm toward the null hypothesis.

It is likely that not all clinicians will be willing to participate in the trial or have their discussions with the patient to be video recorded. However, in previous (14) and ongoing trials over 90% of the clinicians have agreed to participate. In addition, we have >99% power to detect the degree of difference in patient engagement in the decision making process that has been observed on video-recordings in prior trials, so we anticipate having sufficient power to detect a difference in this outcome measure even if some clinicians do not provide consent to video-recording.

Obtaining head CT scans in children at a very low risk of TBI unnecessarily exposes children to the potentially harmful effects of radiation and increases healthcare utilization. Developing and testing the effect of a patient-centered decision aid, Head CT Choice, in a pragmatic multicenter clinician-level parallel randomized clinical trial has the potential to more closely tailor CT use to risk-informed parental preferences and improve the experience of care for parents of children with minor head trauma in the context of a busy and overcrowded ED care delivery system.

## Trial status

Enrollment started April 1, 2014 and we anticipate enrollment will be complete in March 2016.

## Abbreviations

ciTBI: Clinically-important traumatic brain injury; CT: Computed tomography; DCS: Decisional conflict scale; DSMB: Data safety and monitoring board; ED: Emergency department; GCS: Glasgow coma scale; IRB: Institutional review board; MRI: Magnetic resonance imaging; OPTION: Observing patient involvement in decision-making; PAC: Patient advisory council; PECARN: Pediatric emergency care applied research network; PSAC: Patient and stakeholder advisory council; SDM: Shared decision-making; TBI: Traumatic brain injury.

## Competing interests

The authors declare that they have no competing interests.

## Authors’ contributions

EH: conceived, designed, and obtained funding for the study, and drafted and provided critical revisions to the manuscript. KW: wrote sections of and provided critical revisions to the manuscript, contributed to the design of the study. AK: contributed to the conception and design of the study and provided critical revisions to the manuscript. JL: contributed to the design of the study and provided critical revisions to the manuscript. PD: contributed to the conception and design of the study, data analysis and interpretation for the development of the PECARN risk calculator from the publicly available dataset, and provided critical revisions to the manuscript. LT: contributed to the design of the study by critically reviewing and refining the decision aid and provided critical revisions to the manuscript. SW: contributed to the design of the study by providing input on outcome classification for cases of potential ciTBI and provided critical revisions to the manuscript. JLH: contributed to the design of the study by participating in the development of the decision aid and provided critical revisions to the manuscript. LP: contributed to the design of the study by developing the standardized data collection forms and surveys and provided critical revisions to the manuscript. AL: contributed to the design of the study by designing and refining the knowledge questions included in the parent post-visit survey and provided critical revisions to the manuscript. JW: contributed to the design of the study by representing a parent’s perspective in developing and refining the decision aid and prioritization of study outcomes and provided critical revisions to the manuscript. KS: contributed to the design of the study by designing and refining the decision aid based on parents’ and other stakeholders’ input and provided critical revisions to the manuscript. NS: contributed to the conception and design of the study by developing the healthcare utilization analysis, drafted sections of the manuscript, and provided critical revisions the manuscript. MB: contributed to the conception and design of the study by developing the statistical analysis plan and study database and provided critical revisions to the manuscript. JH: contributed to the conception and design of the study by developing the statistical analysis plan, drafting portions of the manuscript, and provided critical revisions to the manuscript. VM: contributed to the conception and design of the study by defining key study outcomes and providing insight into how to meaningfully engage parents, parent representatives, and the ED patient advisory council in the study, and provided critical revisions to the manuscript. NK: provided input on study conception and design, ensured accuracy of analysis and interpretation of the PECARN public access dataset in the development of the risk calculator for ciTBI, assisted in iterative refinement of the decision aid, and provided critical revisions to the manuscript. All authors read and approved the final manuscript.

## Supplementary Material

Additional file 1 **Head CT Choice eligibility assessment case report form.** CT, computed tomography; CSF, cerebral spinal fluid; DA, decision aid; ED, Emergency Department; GCS, Glasgow Coma Scale; PECARN, Pediatric Emergency Care Applied Research Network; VP, ventriculoperitoneal. Click here for file

Additional file 2 **The head CT choice decision aid.** The decision aid (DA) first graphically highlights key differences between concussion and traumatic brain injury to frame and focus the discussion on the risk for traumatic brain injury. The DA communicates the child’s risk for traumatic brain injury using a prose description of natural frequencies and a pictogram (for children with a risk of clinically-important brain injury of at least 0.5%, a pictogram of 100 will be used; for children with a risk of clinically-important brain injury of less than 0.5%, a pictogram of 1000 will be used). The second page clearly outlines the decision that is to be made (head CT scan in the ED versus home observation with no CT scan) and educates the parent in what to watch for and what should prompt a return visit to the ED should they, in collaboration with their clinician, opt for home observation. There is also a table that compares the key advantages and disadvantages of each management option (speed of diagnosis, radiation exposure, pharmacological sedation, cost, potential downsides, and the likely duration of waiting in the ED) and invites the parent to circle the issues that are most important to them. Finally, the DA outlines the three management options (head CT scan in the ED, further observation at home, or having the ED clinician decide on the parent’s behalf) and includes check boxes to document the decision along with a reminder to the parent that they will have the opportunity to revisit the decision with their clinician while in the ED. The third and final page includes a space to provide a contact phone number should the parent need to contact a healthcare provider after leaving the ED. Click here for file

Additional file 3 Parent pre-encounter numeracy and decision-making preferences survey. Click here for file

Additional file 4 Parent post-encounter survey. Click here for file

Additional file 5 Patient healthcare services diary. Click here for file

Additional file 6 Clinician post-encounter survey. Click here for file
