# Supplementary material for: Effectiveness of the head CT choice decision aid in parents of children with minor head trauma: study protocol for a multicenter randomized trial
Source: Trials. 2014 Jun 25;15:253. doi: 10.1186/1745-6215-15-253 (PMC4081461; doi:10.1186/1745-6215-15-253)
Supplement: Additional file 1 — Head CT Choice eligibility assessment case report form. CT, computed tomography; CSF, cerebral spinal fluid; DA, decision aid; ED, Emergency Department; GCS, Glasgow Coma Scale; PECARN, Pediatric Emergency Care Applied Research Network; VP, ventriculoperitoneal. [file 1745-6215-15-253-S1.docx]

**Head CT Choice: Case Report Form**

**Initial Patient Screening**

Date: _______________ Study ID: _______________ CRC Initials: ______________

**INCLUSION CRITERIA: Eligible patients MUST MEET ALL 3 Inclusion Criteria below. (To continue, all three inclusion criteria must be marked “YES”)**

| **Inclusion Criteria** | **YES** | **NO** |
| --- | --- | --- |
| Less than (<)18 years of age |  |  |
| ≤ 24 hours since injury resulting in head trauma |  |  |
| Positive for 1 or 2 of the PECARN criteria (see back) |  |  |

**EXCLUSION CRITERIA: Meets NONE of the Exclusion Criteria below (To continue, all exclusion criteria must be marked “NO”).**

| **Exclusion Criteria** | **YES** | **NO** |
| --- | --- | --- |
| GCS < 15 |  |  |
| Evidence of penetrating trauma, signs of basilar skull fracture or depressed skull fracture on physical examination |  |  |
| Brain tumor |  |  |
| Ventricular shunt |  |  |
| Bleeding disorder |  |  |
| Pre-existing neurological disorders complicating assessment |  |  |
| Syncope or seizure preceded (led to) head trauma |  |  |
| Neuroimaging at an outside hospital before transfer |  |  |
| Signs of altered mental status (agitation, somnolence, repetitive questioning, or slow response to verbal communication) |  |  |
| Known to be pregnant |  |  |
| Communication barriers such as visual or hearing impairment that may preclude use of the decision aid |  |  |

**PECARN CRITERIA: Meets ONE OR TWO of the Exclusion Criteria below:**

**For children YOUNGER THAN 2 YEARS: Circle each row below (*signed off by treating clinician*):**

| **Finding *(if findings unknown, patient should be ineligible)*** | | | |
| --- | --- | --- | --- |
| Severe mechanism (PECARN definition)* | Yes | No | Unknown |
| Loss of consciousness ***> 5 seconds*** | Yes | No | Unknown |
| Acting abnormally per parent** | Yes | No | Unknown |
| Initial ED GCS < 15 by treating clinician*** | Yes | No | Unknown |
| Other signs of altered mental status (PECARN definition)**** | Yes | No | Unknown |
| Presence of occipital, temporal or parietal scalp hematoma | Yes | No | Unknown |
| Palpable skull fracture or unclear if skull fracture | Yes | No | Unknown |

**For children 2 YEARS UP TO 18^th^ birthday: Circle each row below (*signed off by treating clinician*):**

| **Finding *(if findings unknown, patient should be ineligible)*** | | | |
| --- | --- | --- | --- |
| Severe mechanism (PECARN definition)* | Yes | No | Unknown |
| ***Any*** loss of consciousness | Yes | No | Unknown |
| Any vomiting since injury | Yes | No | Unknown |
| Severe headache in ED***** | Yes | No | Unknown |
| Initial ED GCS < 15 by treating clinician*** | Yes | No | Unknown |
| Other signs of altered mental status (PECARN definition)**** | Yes | No | Unknown |
| Any sign of basilar skull fracture****** | Yes | No | Unknown |

******Severe mechanism of injury****:* *motor vehicle crash with patient ejection, death of another passenger, or rollover; pedestrian or bicyclist without helmet struck by a motorized vehicle; falls of more than 0.9 m (3 feet) if < 2 years of age or more than 1.5 m [5 feet] if 2-18 years of age.*

*****Acting abnormally per parent****:* *whether the patient is at his/her baseline or not*

******Other signs of altered mental status****:* *agitation, somnolence, repetitive questioning, or slow response to verbal communication.*

******Initial GCS < 15**: *See table below*

| Current Glasgow Coma Score (Circle 1 number in each column. For infants ≤ 2 years, use the description in parentheses | | | | | |
| --- | --- | --- | --- | --- | --- |
| Eye |  | Verbal |  | Motor |  |
| Spontaneous | 4 | Oriented (coos/babbles) | 5 | Follow commands (spontaneous movement) | 6 |
| Verbal | 3 | Confused (irritable/cries) | 4 | Localizes pain (withdraws to touch) | 5 |
| Pain | 2 | Inappropriate words (cries to pain) | 3 | Withdraws to pain | 4 |
| None | 1 | Incomprehensible sounds (moans) | 2 | Abnormal Flexure posturing | 3 |
|  |  | None | 1 | Abnormal Extension posturing | 2 |
|  |  |  |  | None | 1 |

(Resident or fellow evaluating patient will provide initial assessment of GCS, to be confirmed by attending/consultant after randomization but prior to delivery of the DA or discussion with the parent)

*******Severe headache**: Intense

Mild: refers to a headache that is barely noticeable or one that the patient does not complain about

Moderate: In between mild and severe

Unclear: Patient cannot specify the intensity of the headache

********Any sign of basilar skull fracture**: includes hemotympanum, CSF rhinorrhea, CSF otorrhea, or periorbital ecchymoses (Raccoon eyes)

**Clinician Sign-off**

___________________________________ ___________________________________ __________________

Printed Name Signature Date

**Consent**

Date of registration: __________________________________

(Date informed consent was signed)

Arm: □ Usual Care

□ Decision Aid

(This is the arm the treating clinician is assigned to.)

Other: __________________________________

(Field for any additional information the study coordinator feels is relevant)

Patient found to be ineligible after consent: □ No

□ Yes

If yes, provide reason in text box below:

Ineligibility criteria found: __________________________________

**Patient Demographics**

Medical record number: __________________________________

Patient first name: __________________________________

Patient middle initial: __________________________________

Patient last name: __________________________________

Gender of child: □ Male

□ Female

Date of birth: __________________________________

Age of child: __________________________________

Age Group: □ < 2 years old

□ 2+ years old

Insurance: □ Government (Medicare, Medicaid, Veterans Health

(the patient's primary insurance Administration, DOD)

payer for the ED visit) □ Commercial (all fee for service carriers and PPO's)

□ HMO (coverage that provides healthcare services

for members on a prepaid basis)

□ None

**Legal Guardian/Parent Information**

Guardian/Parent's principal language: □ English

□ Spanish

□ Russian

□ French

□ Chinese

□ Unknown

□ Other

Other language:________________________________

Guardian/Parent's preferred secondary □ Secondary Phone

means of contact: (The first means of □ Mail

contact for all guardian/parents is his/her □ E-Mail

primary phone number)

Best Phone Number to reach Guardian/Parent: __________________________________

(cell or other)

Guardian/Parent Secondary Phone Number: __________________________________

(home or other)

Guardian/Parent E-mail address: __________________________________

Home Address: __________________________________

(Street, City, State and Zip)

**General Information**

Who was present: □ Mother

□ Father

□ Other Family Member

□ Caregiver

□ Friend

Was the encounter recorded (video or audio): □ No

□ Yes - Video and Audio

□ Yes - Audio only

Why was encounter not recorded: □ Provider declined

□ Patient declined

□ Video equipment not available/functional

□ Other

Other reason for encounter not recorded:

__________________________________

Clinical evidence of other (non-head) □ No

substantial injuries: □ Yes

(could include: fractures, intra-abdominal

injuries, intra-thoracic injuries, and

lacerations requiring operating room repair)

Injury location: □ Extremity

□ C-spine

□ Chest/back/flank

□ Intra-abdominal

□ Pelvis

□ Laceration requiring repair in OR

□ Other

Other injury location: ____________________________

□ Unable to fully assess

**Course in ED**

Date & time of injury: __________________________________

Date and time of registration in ED: __________________________________

Date & time of discharge from ED: __________________________________

Was patient observed in the ED after □ No

initial ED evaluation to determine □ Yes

whether to obtain a CT:

Prior to CT or if no CT obtained, □ Patient never had a headache

the patient's headache: □ Resolved without any analgesia

□ Resolved with analgesia

□ Improved (but did not resolve)

□ Stayed the same

□ Worsened

□ Can't assess - patient preverbal or nonverbal

□ Did not reassess

Prior to CT or if no CT obtained, □ Patient never vomited

the patient's vomiting: □ Resolved without meds

□ Resolved with meds

□ Continued (stayed the same)

□ Worsened

□ Did not reassess

Prior to CT or if no CT obtained, □ Patient never had GCS < 15

the patient's mental status/GCS: □ Improved to normal (GCS 15)

□ Improved but not to normal

□ Stayed the same

□ Worsened

□ Did not reassess

Patient Comments: __________________________________

(Any comments patient made that are pertinent to study)
