# Supplementary material for: Effectiveness of the head CT choice decision aid in parents of children with minor head trauma: study protocol for a multicenter randomized trial
Source: Trials. 2014 Jun 25;15:253. doi: 10.1186/1745-6215-15-253 (PMC4081461; doi:10.1186/1745-6215-15-253)
Supplement: Additional file 2 — The head CT choice decision aid. The decision aid (DA) first graphically highlights key differences between concussion and traumatic brain injury to frame and focus the discussion on the risk for traumatic brain injury. The DA communicates the child’s risk for traumatic brain injury using a prose description of natural frequencies and a pictogram (for children with a risk of clinically-important brain injury of at least 0.5%, a pictogram of 100 will be used; for children with a risk of clinically-important brain injury of less than 0.5%, a pictogram of 1000 will be used). The second page clearly outlines the decision that is to be made (head CT scan in the ED versus home observation with no CT scan) and educates the parent in what to watch for and what should prompt a return visit to the ED should they, in collaboration with their clinician, opt for home observation. There is also a table that compares the key advantages and disadvantages of each management option (speed of diagnosis, radiation exposure, pharmacological sedation, cost, potential downsides, and the likely duration of waiting in the ED) and invites the parent to circle the issues that are most important to them. Finally, the DA outlines the three management options (head CT scan in the ED, further observation at home, or having the ED clinician decide on the parent’s behalf) and includes check boxes to document the decision along with a reminder to the parent that they will have the opportunity to revisit the decision with their clinician while in the ED. The third and final page includes a space to provide a contact phone number should the parent need to contact a healthcare provider after leaving the ED. [file 1745-6215-15-253-S2.pdf]

Let's talk about concussion and your child's risk for more serious injury such as bleeding in or around the brain.

Concussion

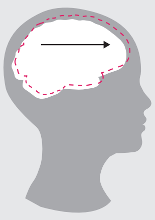

- Symptoms may include headache, nausea, dizziness, or difficulty concentrating
- Symptoms should resolve in several days to a few months
- Recovery is almost always complete
- Cannot be seen on a CT scan

Brain Injury

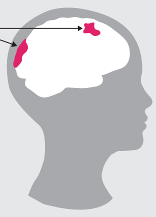

- Occurs when the head injury is severe enough to cause bleeding in or around the brain
- May require medical intervention such as a stay in the hospital or surgical procedure

In 100 children with minor head injury similar to your child:

1 will have brain injury and **99 will not**

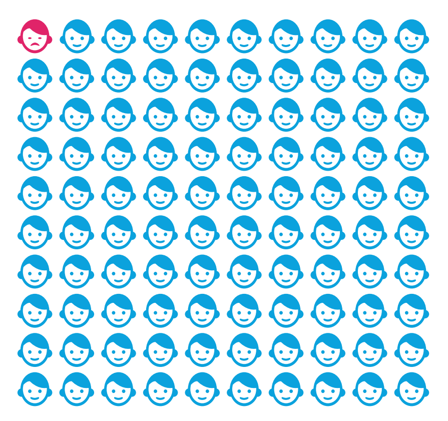

Kuppermann et al., Lancet, 2009

After monitoring your child in the emergency department for a period of time, we will find out if there is any serious bleeding in or around the brain with:

HEAD CT SCAN

You can have a head CT scan test done to determine if your child has had a brain injury.

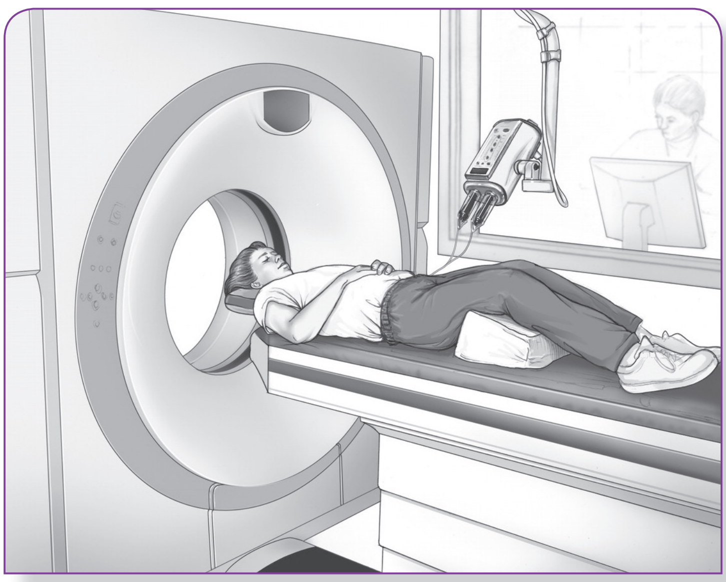

or

OBSERVATION AT HOME

If your child's symptoms are the same or better in the next 1-2 days, then there was no serious bleeding in or around the brain.

It is very unlikely, but if your child develops new or worsening symptoms such as these, bring him/her back to the Emergency Department as soon as possible.

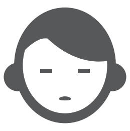

Lack of alertness (if they are becoming less and less alert within the next day)

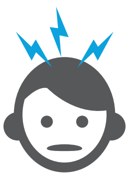

Severely worsening headache (despite resting)

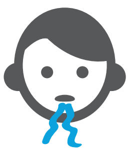

Vomiting (enough episodes to interfere with eating)

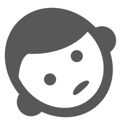

Unsteady or cannot walk

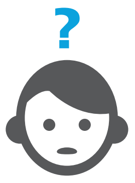

Difficulty talking or recognizing people

Your child can maintain regular activities such as sleep.

Please circle the issues that are most important to you and your child.

	SPEED OF DIAGNOSIS	RADIATION	SEDATION	COST	POTENTIAL DOWNSIDES	WAIT IN ED
**HEAD CT SCAN**	Now	Yes	Possible	May increase cost depending on your coverage	May find irrelevant things that lead to more tests	Typically longer
**OBSERVATION AT HOME**	Delayed	No	No	No added cost	Potential return to ED if symptoms worsen	Typically shorter

After discussing this together, we want to do:

- ☐ HEAD CT SCAN
- ☐ OBSERVATION AT HOME
- ☐ Let the Emergency Department doctor decide what to do next

You will have the opportunity to revisit this decision with your doctor while you are in the Emergency Department.

For questions and concerns, please contact:

____

____

Pediatric Head CT Choice: Version 28-1/100

©2014 Mayo Foundation for Medical Education and Research. All rights reserved. Revised 3/14/2014
