# Supplementary material for: Effectiveness of the head CT choice decision aid in parents of children with minor head trauma: study protocol for a multicenter randomized trial
Source: Trials. 2014 Jun 25;15:253. doi: 10.1186/1745-6215-15-253 (PMC4081461; doi:10.1186/1745-6215-15-253)
Supplement: Additional file 3 — Parent pre-encounter numeracy and decision-making preferences survey. [file 1745-6215-15-253-S3.doc]

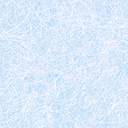


Shared Decision Making in Parents of Children with Minor Head Injury

**Pre Encounter Survey**

Study ID:


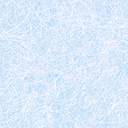


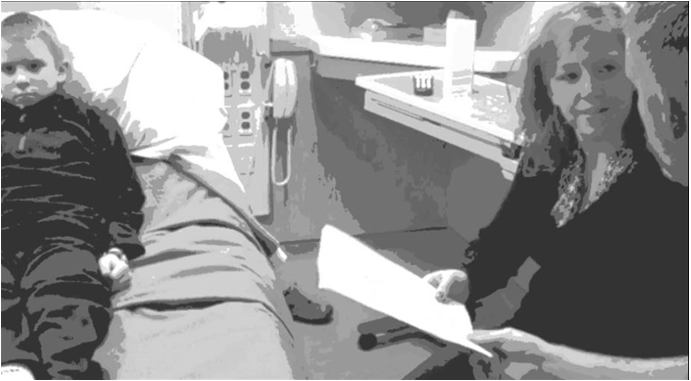


**Today’s Date:** _ _ / _ _ / _ _ _ _

MM DD YYYY

Thank you for helping with this study. Your answers are important to us. Please take the time to read and answer each question carefully by marking the box that best represents your answer. Some items ask you to fill in the blank space provided for your answer. Your responses are confidential and your clinician will not see your answers. After completing this survey, please return it to the study coordinator.

1. For each of the following questions, please check the box that best reflects how good you are at doing the following things:

| How good are you at… Not good  at all | | |  |  | Extremely  good | |
| --- | --- | --- | --- | --- | --- | --- |
| working with fractions?. . . . . . . . . . | 1 | 2 | 3 | 4 | 5 | 6 |
| working with percentages?. . . . . . . . | 1 | 2 | 3 | 4 | 5 | 6 |
| calculating a 15% tip?. . . . . . . . . . . . | 1 | 2 | 3 | 4 | 5 | 6 |
| figuring out how much a shirt will cost if it is 25% off?. . . . . . . . . . . .. . | 1 | 2 | 3 | 4 | 5 | 6 |

1. For each of the following questions, please check the box that best describes your answer.
   1. When reading the newspaper, how helpful do you find tables and graphs that are parts of a story?

| Not helpful  at all |  |  |  |  | Extremely  helpful |
| --- | --- | --- | --- | --- | --- |
| 1 | 2 | 3 | 4 | 5 | 6 |

- 1. When people tell you the chance of something happening, do you prefer that they use words (“it rarely happens”) or numbers (“there’s a 1% change”)?

| Always prefer  words | |  |  | Always prefer  numbers | |
| --- | --- | --- | --- | --- | --- |
| 1 | 2 | 3 | 4 | 5 | 6 |
|  |  |  |  |  |  |

- 1. When you hear a weather forecast, do you prefer predictions using percentages (eg, “there will be a 20% chance of rain today”) or predictions using only words (eg, “there is a small chance of rain today”)?

| Always prefer  percentages | |  |  | Always prefer  words | |
| --- | --- | --- | --- | --- | --- |
| 1 | 2 | 3 | 4 | 5 | 6 |

- 1. How often do you find numerical information to be useful?

| Never |  |  |  |  | Very often |
| --- | --- | --- | --- | --- | --- |
| 1 | 2 | 3 | 4 | 5 | 6 |

1. How often do you have someone help you read instructions, pamphlets, or other written material from your doctor or pharmacy?

| 1 Never |
| --- |
| 2 Occasionally |
| 3 Sometimes |
| 4 Often |
| 5 Always |

1. How confident are you at filling out medical forms by yourself?

| 1 Never |
| --- |
| 2 Occasionally |
| 3 Sometimes |
| 4 Often |
| 5 Always |

1. How often do you have problems learning about your medical condition because of difficulty understanding written information?

| 1 Never |
| --- |
| 2 Occasionally |
| 3 Sometimes |
| 4 Often |
| 5 Always |

1. What is the highest level of schooling you have completed?

| 1 Some high school or less |
| --- |
| 2 High school graduate or GED |
| 3 Some college or associate’s degree (including community college) or  vocational technical, or business school degree |
| 4 Four-year college graduate (bachelor’s degree) |
| 5 Graduate or professional school degree |
| 6 Other, please specify: ____________________________________ |

1. Which of the following categories best describes your household income last year?

| 1 Less than $20,000 |
| --- |
| 2 $20,000 to $29,999 |
| 3 $30,000 to $39,999 |
| 4 $40,000 to $59,999 |
| 5 $60,000 to $79,999 |
| 6 $80,000 to $99,999 |
| 7 $100,000 or more |

Please indicate who completed the questions in this booklet. (Mark one.)

| 1 | Child's Mother |
| --- | --- |
| 2 | Child's Father |
| 3 | Another family or household member |
| 4 | Friend of the family |
| 5 | Clinic staff |
| 6 | Other, please specify: _____________________________________ |

Thank you again for your contribution!

Please return the survey to the study coordinator.
