# Supplementary material for: Effectiveness of the head CT choice decision aid in parents of children with minor head trauma: study protocol for a multicenter randomized trial
Source: Trials. 2014 Jun 25;15:253. doi: 10.1186/1745-6215-15-253 (PMC4081461; doi:10.1186/1745-6215-15-253)
Supplement: Additional file 4 — Parent post-encounter survey. [file 1745-6215-15-253-S4.doc]

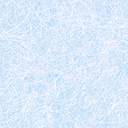


Study ID:

Shared Decision Making

in Parents of Children with Head Trauma

**Post Encounter Survey**


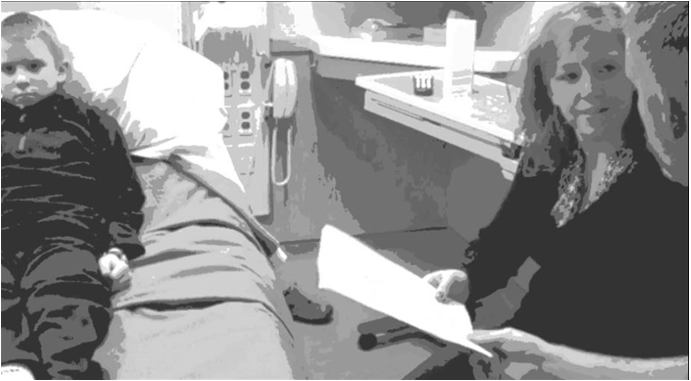


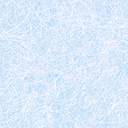


Version 100

**Today’s Date**: _ _ / _ _ / _ _ _ _

Month Day Year

| **Thank you for helping with this study. Your answers are very important to us. Please take the time to read and answer each question. Your responses are confidential and your clinician will not see your answers.** |
| --- |

| **1. Which of the following options of care best describes the decision you made today**  **regarding your child receiving a Head CT?** |
| --- |

| 1 | To have a Head CT |
| --- | --- |
| 2 | Active observation at home |
| 3 | To have the emergency doctor make the decision for me |

| **2. The following questions are about the visit you had with your clinician today and the discussion you had about whether your child should receive a Head CT. Please mark the best answer to each of these questions by marking an X in the box you select.** |
| --- |

| a. How would you describe the ***amount of information*** provided to you about your child receiving a Head CT during the visit? |
| --- |

| Too  little  information | ----------------------------------- | | Just the right  amount of  information | ---------------------------------- | | Too  much information |
| --- | --- | --- | --- | --- | --- | --- |
| 1 | 2 | 3 | 4 | 5 | 6 | 7 |

| b. How would you describe the ***clarity of information*** provided to you about whether your child should receive a Head CT during the visit? |
| --- |

| Not clear  at all | ----------------------------------- | | Somewhat clear | ----------------------------------- | | Extremely  clear |
| --- | --- | --- | --- | --- | --- | --- |
| 1 | 2 | 3 | 4 | 5 | 6 | 7 |

Page 1

| c. How ***helpful*** was the information provided to you about whether your child should receive a Head CT during the visit? |
| --- |

| Not helpful at all | ----------------------------------- | | Somewhat  helpful | ----------------------------------- | | Extremely  helpful |
| --- | --- | --- | --- | --- | --- | --- |
| 1 | 2 | 3 | 4 | 5 | 6 | 7 |

| d. Would you want to get information about other options for your child’s care in the same way that you got information about whether your child should receive a Head CT during the visit? |
| --- |

| Yes, for  sure | ----------------------------------- | | Not  sure | ----------------------------------- | | No, not  at all |
| --- | --- | --- | --- | --- | --- | --- |
| 1 | 2 | 3 | 4 | 5 | 6 | 7 |

| e. Would you recommend the way that you and your provider shared information about whether your child should receive a Head CT to other patients? |
| --- |

| Yes, I  strongly  recommend  it | ----------------------------------- | | Not sure  whether to recommend it or not | ----------------------------------- | | No, I  strongly recommend against it |
| --- | --- | --- | --- | --- | --- | --- |
| 1 | 2 | 3 | 4 | 5 | 6 | 7 |

Page 2

**Please answer the following questions as best you can.**

**This is not a test – what is important is that your answers show what you think.**

| **3. Below are listed some statements about brain injury and Head CT. Please make an ‘x’ inside that box to let us know whether you think they are true, false, or you are unsure.** | True | False | Unsure |
| --- | --- | --- | --- |
| 1. There is a possibility that my child could have   bleeding in or around his/her brain. | 1 | 2 | 3 |
| 1. Having a head CT scan is the only option that I   have to know if my child has a brain injury. | 1 | 2 | 3 |
| 1. A head CT scan is necessary to diagnose a   concussion. | 1 | 2 | 3 |
| 1. A brain injury always requires a medical   intervention. | 1 | 2 | 3 |
| 1. Having a head CT scan will confirm right away if   my child has a brain injury. | 1 | 2 | 3 |
| 1. My child will not be exposed to radiation with a   head CT scan. | 1 | 2 | 3 |
| 1. I only need to return to the Emergency Department (ED) if my child is getting worse in the next 12 hours following our discharge from the ED. | 1 | 2 | 3 |
| 1. The CT scan may find irrelevant things that will lead to more tests. | 1 | 2 | 3 |
| 1. If my child vomits but is still able to eat, I should return to the Emergency Department. | 1 | 2 | 3 |
| 1. I should keep my child awake for 12 hours after we leave the Emergency Department, to make sure they are okay. | 1 | 2 | 3 |

| **4. How many children like your child do you think will have significant brain injury**  **out of 100 children?** | | | | | |
| --- | --- | --- | --- | --- | --- |
|  | | | | | |
|  | Provide a value of 0-100 or respond ‘I do not know’: | | | | |
|  | | | | | |
|  | |  |  |  | I do not know. |

Page 3

| **5. Thinking about the conversation that you had with your child’s clinician today**  **about whether or not your child should have a Head CT, please mark an x inside the**  **box that best describes your agreement with the following statements.** | | | | | |
| --- | --- | --- | --- | --- | --- |
|  | Strongly  disagree | Disagree | Neither  agree nor  disagree | Agree | Strongly  agree |
| a. I know which options are available to me. | 1 | 2 | 3 | 4 | 5 |
| b. I know the benefits of each option. | 1 | 2 | 3 | 4 | 5 |
| c. I know the risks and side effects of each  option. | 1 | 2 | 3 | 4 | 5 |
| d. I am clear about which benefits matter  most to me. | 1 | 2 | 3 | 4 | 5 |
| e. I am clear about which risks and side  effects matter most to me. | 1 | 2 | 3 | 4 | 5 |
| f. I am clear about which is more important  to me (the benefits or the risks and side  effects). | 1 | 2 | 3 | 4 | 5 |
| g. I have enough support from others to  make a choice. | 1 | 2 | 3 | 4 | 5 |
| h. I am choosing without pressure from others. | 1 | 2 | 3 | 4 | 5 |
| i. I have enough advice to make a choice. | 1 | 2 | 3 | 4 | 5 |
| j. I am clear about the best choice for my child. | 1 | 2 | 3 | 4 | 5 |
| k. I feel sure about what to choose. | 1 | 2 | 3 | 4 | 5 |
| l. This choice is easy for me to make. | 1 | 2 | 3 | 4 | 5 |
| m. I feel I have made an informed choice. | 1 | 2 | 3 | 4 | 5 |
| n. My choice shows what is important to me. | 1 | 2 | 3 | 4 | 5 |
| o. I expect to stick with my choice. | 1 | 2 | 3 | 4 | 5 |
| p. I am satisfied with my choice. | 1 | 2 | 3 | 4 | 5 |

Page 4

| **6. How much do you trust the clinician who discussed your child’s receiving a Head**  **CT during your visit today to:** | | | | | | | | |  |
| --- | --- | --- | --- | --- | --- | --- | --- | --- | --- |
|  | Not at all | A little | Somewhat | | | Mostly | Completely | | |
|  |  |  | |  |  | | |  |  |
| a. Always tell you the truth. | 1 | 2 | | 3 | 4 | | | 5 |  |
| b. Provide you with accurate, up-to-date,  medical information. | 1 | 2 | | 3 | 4 | | | 5 |  |
| c. Make it easy for you to bring up a prior  discussion about your condition and  discuss it again. | 1 | 2 | | 3 | 4 | | | 5 |  |
| d. Make excellent medical judgments on  your behalf. | 1 | 2 | | 3 | 4 | | | 5 |  |
| e. Do everything medically that should be  done in order to ensure the best possible  result. | 1 | 2 | | 3 | 4 | | | 5 |  |
| f. Tell you when you could benefit from  seeing a specialist. | 1 | 2 | | 3 | 4 | | | 5 |  |
| g. Tell you if a mistake was made about your  treatment. | 1 | 2 | | 3 | 4 | | | 5 |  |
| h. Put your medical needs above all other  considerations, including cost. | 1 | 2 | | 3 | 4 | | | 5 |  |
| i. Listen well so he/she understands your  needs and concerns. | 1 | 2 | | 3 | 4 | | | 5 |  |
| j. Never pretend to know things when he/she  is not sure. | 1 | 2 | | 3 | 4 | | | 5 |  |

| **7. Thinking about the decision you made today about your child receiving a Head CT,**  **would your decision be different if your care was free (no cost to you or your insurer)?** |
| --- |

| 1 | Yes |
| --- | --- |
| 2 | No |

Page 5

| **8. During visits where a decision is made with a clinician about care for my child, I am**  **most comfortable when…** |
| --- |

|  | | | | |
| --- | --- | --- | --- | --- |
| 1 | 2 | 3 | 4 | 5 |

| **9. Sometimes people need help completing surveys. Please indicate who answered the**  **majority of the questions in this booklet. (Mark one.)** |
| --- |

| 1 | Child's Mother |
| --- | --- |
| 2 | Child's Father |
| 3 | Another family or household member |
| 4 | Friend of the family |
| 5 | Clinic staff |
| 6 | Other, please specify: _____________________________________ |

Thank you for completing the survey!

Please return it to the study coordinator.

Page 6
