# Supplementary material for: Effectiveness of the head CT choice decision aid in parents of children with minor head trauma: study protocol for a multicenter randomized trial
Source: Trials. 2014 Jun 25;15:253. doi: 10.1186/1745-6215-15-253 (PMC4081461; doi:10.1186/1745-6215-15-253)
Supplement: Additional file 5 — Patient healthcare services diary. [file 1745-6215-15-253-S5.doc]

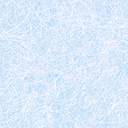


Shared Decision Making

in Parents of Children with Head Trauma

Use of Healthcare Services

Diary


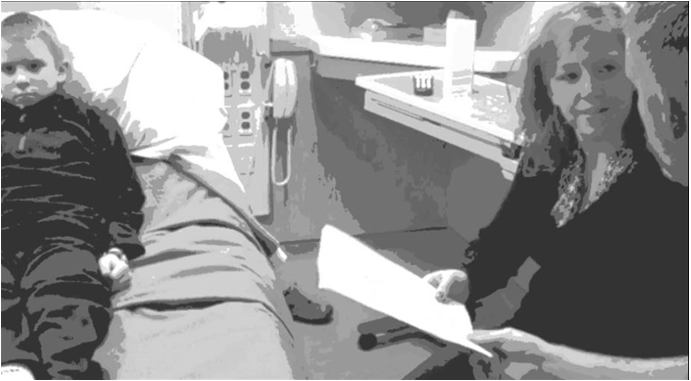


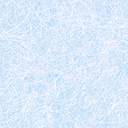


You were recently enrolled in the **Head CT Choice Study.** As part of this study, we would like to understand your use of healthcare services in the **7-days AFTER discharge** from the emergency department.

This *Diary* is for you to keep, in order that you may have a record of events related to your child’s health from the time of your discharge from the emergency department.

In addition, as you may be aware, a study researcher will contact you in about 7-days after you joined the study to ask you about events related to your child’s health.

To keep track of these events you may find this *Diary* will help you answer the questions. If you require additional space to record details of health service use, please use the sheet entitled *Events Diary - Additional Information* that is included.

**Hospital Admissions**

If your child is admitted to a hospital, please write the **name** of the hospital, **reason** for being admitted, **dates** of each admission and discharge. Use one line for each hospital admission.

| **Name of Hospital** | **Reason for Admission** | **Date of Admission** | **Date of Discharge** |
| --- | --- | --- | --- |
|  |  |  |  |
|  |  |  |  |
|  |  |  |  |

**Emergency Department Visits**

If your child visits an emergency department, please write the **name** of the hospital, **reason** for the visit and **date** of each visit. Use one line for each Emergency Department visit.

| **Name of Hospital** | **Reason for visit** | **Date of**  **Visit** |
| --- | --- | --- |
|  |  |  |
|  |  |  |
|  |  |  |

**Physician Office Visit**

If your child visits a physician’s office, please write the **name** of the health care facility, **reason** for the visit, **type** of physician and **date** of each visit. Use one line for each office visit.

| **Name of Clinic or Physician Office** | **Reason for Visit** | **Type of Physician**  **(Please Check)** | **Date of Visit** |
| --- | --- | --- | --- |
|  |  | ☐ Pediatrician/Family Practice/Primary Care Clinician  ☐ Neurologist  ☐ Other_________________ |  |
|  |  | ☐ Pediatrician/Family Practice/Primary Care Clinician  ☐ Neurologist  ☐ Other_________________ |  |
|  |  | ☐ Pediatrician/Family Practice/Primary Care Clinician  ☐ Neurologist  ☐ Other_________________ |  |

**Testing or Procedures**

In the next section, please document any testing that your child received in the 7-days after leaving the emergency department., including **X-rays, CTs, MRIs or neurological testing**. Please check in the boxes below any tests that were obtained,, the name of the hospital or clinic where the test was done ,and the date of the test.

| **Type of Test or Procedure** | **Name of Hospital or Clinic** | **Date of Test** |
| --- | --- | --- |
| ☐ X-ray  ☐ CT (Computed Tomography)  ☐ MRI (Magnetic Resonance Imaging)  ☐ Intubation (breathing tube in lungs)  ☐ Emergency brain surgery  ☐ Other (describe):__________________________ |  |  |
| ☐ X-ray  ☐ CT (Computed Tomography)  ☐ MRI (Magnetic Resonance Imaging)  ☐ Intubation (breathing tube in lungs)  ☐ Emergency brain surgery  ☐ Other (describe):__________________________ |  |  |
| ☐ X-ray  ☐ CT (Computed Tomography)  ☐ MRI (Magnetic Resonance Imaging)  ☐ Intubation (breathing tube in lungs)  ☐ Emergency brain surgery  ☐ Other (describe):__________________________ |  |  |

***Events Diary - Additional Information***
