# Supplementary material for: Effectiveness of the head CT choice decision aid in parents of children with minor head trauma: study protocol for a multicenter randomized trial
Source: Trials. 2014 Jun 25;15:253. doi: 10.1186/1745-6215-15-253 (PMC4081461; doi:10.1186/1745-6215-15-253)
Supplement: Additional file 6 — Clinician post-encounter survey. [file 1745-6215-15-253-S6.doc]

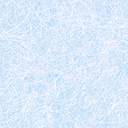


Patient Study ID:

Clinician’s Initials:

Shared Decision Making

in Parents of Children with Head Trauma

**Clinician Survey**


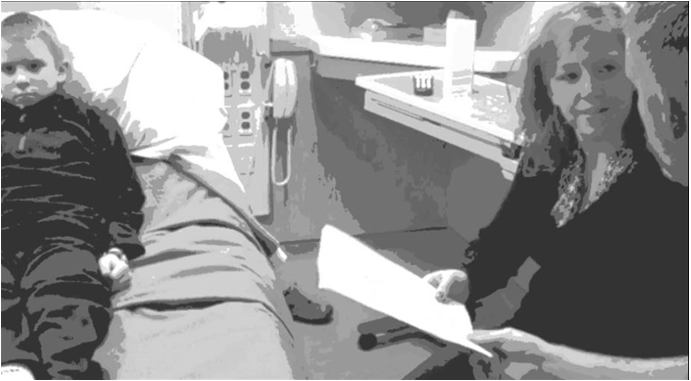


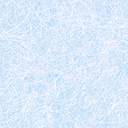


**Today’s Date**: _ _ / _ _ / _ _ _ _

Month Day Year

| **1. In the clinical encounter where the decision was made with the parent(s) (check one box):** |
| --- |

| 1 | I made the decision on my own. |
| --- | --- |
| 2 | I made the decision after seriously considering the parent’s opinion. |
| 3 | The parent(s) and I shared the responsibility for making the decision after considering both of our opinions. |
| 4 | The parent(s) made the decision after seriously considering my opinion. |
| 5 | The parent(s) made the decision on his/her/their own. |

| **2. You gave information about pediatric head trauma, the child’s risk for traumatic brain injury (TBI) in need of acute intervention (neurosurgical intervention, elevation of depressed skull fracture, intubation for TBI > 24hrs, or hospitalization for TBI 2 nights or more), and their diagnostic options during this visit. How *helpful* do you think this information was to the parent(s)?** |
| --- |

| Not helpful at all | ----------------------------------- | | Somewhat  helpful | ----------------------------------- | | Extremely  helpful |
| --- | --- | --- | --- | --- | --- | --- |
| 1 | 2 | 3 | 4 | 5 | 6 | 7 |

| **3. Would you want to present information about other diagnostic choices in the same way that you presented information about pediatric Head CT during this visit?** |
| --- |

| Yes, for  sure | ----------------------------------- | | Not  sure | ----------------------------------- | | No, not  at all |
| --- | --- | --- | --- | --- | --- | --- |
| 1 | 2 | 3 | 4 | 5 | 6 | 7 |

Page 1

| **4. Would you *recommend* to other providers the way that you presented information on pediatric head trauma, the child’s risk for TBI in need of acute intervention (neurosurgical intervention, elevation of depressed skull fracture, intubation for TBI > 24hrs, hospitalization for TBI 2 nights or more), and their diagnostic options during this visit?** |
| --- |

| Yes, I would strongly  recommend  it | ----------------------------------- | | Not sure  whether to recommend it or not | ----------------------------------- | | No, I would  strongly recommend against it |
| --- | --- | --- | --- | --- | --- | --- |
| 1 | 2 | 3 | 4 | 5 | 6 | 7 |

| **5. Thinking about the conversation you had with the parents, the child’s risk for**  **TBI in need of acute intervention, and their diagnostic options during this visit, please place an “X” inside the box that best describes your agreement with the following statements.** | | | | | |
| --- | --- | --- | --- | --- | --- |
|  | Strongly agree | Agree | Neither agree nor disagree | Disagree | Strongly disagree |
|  |  |  |  |  |  |
| a. I feel the parent(s) has/have made a  choice informed by the information  we discussed. . . . . . . . . . . . . . . | 1 | 2 | 3 | 4 | 5 |
| b. The parent’s decision shows what is  important to him/her. . . . . . . . . | 1 | 2 | 3 | 4 | 5 |
| c. I expect the parent(s) to stick with  his/her decision. . . . . . . . . . . . . | 1 | 2 | 3 | 4 | 5 |
| d. I think the parent is satisfied with  his/her decision. . . . . . . . . . . . . | 1 | 2 | 3 | 4 | 5 |

| **6. What is the level of suspicion for the presence of TBI, regardless of whether a CT is**  **being ordered or obtained (intracranial hematoma, cerebral contusion, cerebral**  **edema or depressed skull fracture; excludes isolated linear skull fracture)?** |
| --- |

| 1 | 2 | 3 | 4 | 5 |
| --- | --- | --- | --- | --- |
| **< 1 %** | **1-5%** | **6-10%** | **11-50%** | **> 50%** |

| **7. What is the level of suspicion of TBI in need of acute intervention, regardless of**  **whether a CT is being ordered or obtained (neurosurgical intervention, elevation of depressed skull fracture, intubation for TBI > 24hrs, hospitalization for TBI 2 nights**  **or more)?** |
| --- |

| 1 | 2 | 3 | 4 | 5 |
| --- | --- | --- | --- | --- |
| **< 1 %** | **1-5%** | **6-10%** | **11-50%** | **> 50%** |

Page 2

| **8. If a head CT was obtained, rank the top three indications that were most important in influencing your decision to obtain a head CT for this child:** |
| --- |

| 1 | Young age |
| --- | --- |
| 2 | Seizure |
| 3 | Clinical evidence of skull fracture |
| 4 | Skull fracture on x-ray |
| 5 | Mechanism |
| 6 | Headache |
| 7 | Scalp hematoma |
| 8 | Trauma team request |
| 9 | LOC |
| 10 | Vomiting |
| 11 | Neurological deficit (other than mental status) |
| 12 | Referring MD request |
| 13 | Amnesia |
| 14 | Decreased mental status |
| 15 | Parental anxiety / request |
| 16 | Other (describe): ___________________________ |

**Thank you for completing the survey and participation in the trial. Please return the survey to the study coordinator.**
